# *TableButler *– a Windows based tool for processing large data tables generated with high-throughput methods

**DOI:** 10.1186/1471-2105-10-235

**Published:** 2009-07-29

**Authors:** Christian Schwager, Ute Wirkner, Amir Abdollahi, Peter E Huber

**Affiliations:** 1 Department of Radiation Oncology, German Cancer Research Center (DKFZ) and University of Heidelberg Medical School, Heidelberg, Germany; 2 Center of Cancer Systems Biology, Dept. of Medicine, Caritas St. Elizabeth's Medical Center, Tufts University School of Medicine, Boston, Massachusetts, USA

## Abstract

**Background:**

High-throughput "omics" based data analysis play emerging roles in life sciences and molecular diagnostics. This emphasizes the urgent need for user-friendly windows-based software interfaces that could process the diversity of large tab-delimited raw data files generated by these methods. Depending on the study, dozens to hundreds of these data tables are generated. Before the actual statistical or cluster analysis, these data tables have to be combined and merged to expression matrices (e.g., in case of gene expression analysis). Gene annotations as well as information concerning the samples analyzed may be appended, renewed or extended. Often additional data values shall be computed or certain features must be filtered out.

**Results:**

In order to perform these tasks, we have developed a Microsoft Windows based application, "***TableButler***", which allows biologists or clinicians without substantial bioinformatics background to perform a plethora of data processing tasks required to analyze the large-scale data.

**Conclusion:**

***TableButler ***is a monolithic Windows application. It is implemented to handle, join and preprocess large tab delimited ASCII data files. The intuitive user interface enables scientists (e.g. biologists, clinicians or others) to setup workflows for their specific problems by simple drag-and drop like operations.

For more details about ***TableButler***, visit .

## Background

DNA filter- and microarrays are widely used in functional genomics research. Complete genomes can be spotted on such arrays. After hybridization and image analysis large data tables are generated. From each hybridization ten thousands (genome wide expression arrays) to hundred thousands (genome wide filter arrays or CGH microarrays) of data lines for all measured gene features are generated and saved. Data may be saved as structured XML-documents, mostly using well defined and standardized MAGE-ML [1] object model and definitions. This requires subsequent use of programs that can import XML documents (e.g. commercial solutions like Rosetta Resolver [2] or open source tools like Bioconductor [3] based on R package [4]). Alternatively, most programs can generate generic tab-delimited text files, which can easily be imported into nearly any spreadsheet or statistics program or databases. Depending on the study type, dozens to hundreds of these data tables are generated. Before the actual statistical or cluster analysis, these data tables have to be combined and merged to expression matrices, gene annotations or sample informations may be appended, renewed or extended. Often additional data values are to be computed or certain features must be filtered out.

One way to perform such tasks can be the use of commercially available microarray databases with integrated handling and analyses tools (e.g. Rosetta Resolver, Agilent [2]). Large institutes have developed customized solutions (e.g. SMD, Stanford [5]). Alternatively open source solutions (e.g. BASE [6] and JExpress [7] or TM4 [8]) may be setup. However, all such solutions require considerable computer expertise both for the installation set-up and for the system maintenance.

Some of the tasks mentioned above may also be solved with standard spreadsheet programs from office packages (e.g. OpenOffice [8]). Unfortunately, both the commercial as well as the freeware solutions have severe limitations. Data files may not exceed 65000 rows and/or 255 columns and may create bizarre results when using incorrect national settings for number or time formats.

Moreover, one can implement such tools "de novo" (using e.g. Perl [9], C [10] or R [4]), which again requires expert knowledge from bio-informaticians. In fact, this approach requires an installation of the respective development environments and – even more critical – detailed background knowledge and experience on development and optimization of algorithms as well as the implementation of such tasks.

In contrast, our here presented solution, ***TableButler ***is a standalone application (less then 1 Megabyte) which can perform most of the commonly used operations prior to statistical or cluster analysis of microarray data. At present, ***TableButler ***exclusively works with tab-delimited data files, avoiding the need to keep track with file format changes in proprietary spreadsheet formats or varying XML-dialects to enwrap the information. The rich MS Windows user interface allows convenient set-up of operations for non-bioinformatics educated users. By default, all derived data files are generated with new file names, thus preventing data loss due to erroneous actions.

Parameters of interactively set-up filters and operations may be saved and recalled later on for similar operations. This guarantees consistent pre-processing of data tables across project and users.

## Implementation

### File selection

**Figure 1:**
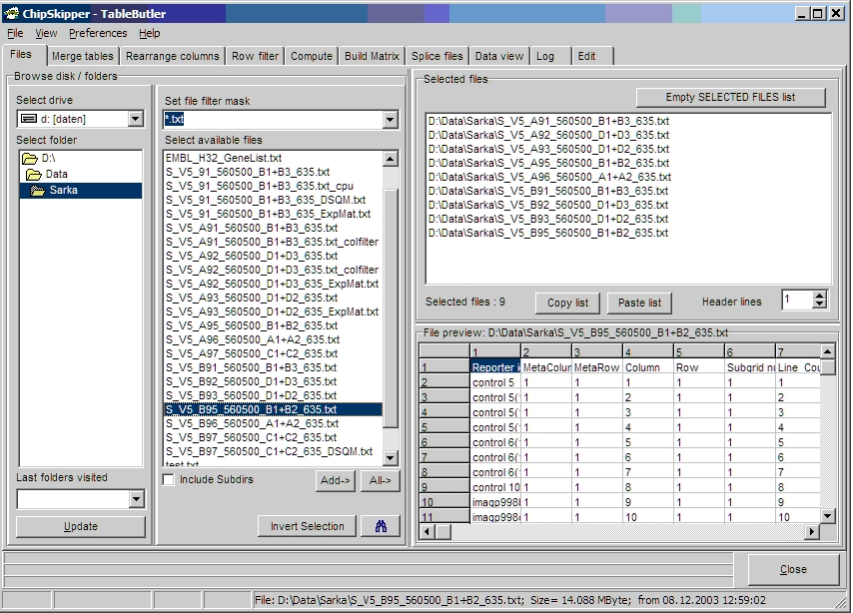
***TableButlers's *file selector**. Left panels: files system browser, Top right: list of selected files. Bottom right: table preview of just selected file.

Multiple files, e.g. primary result tables from single microarray hybridizations, may be selected for batch processing. An "Explorer" like file selector allows selecting files from different folders or drives. Furthermore, all data files with a given file mask from a complete folder tree may be selected with a few mouse clicks. Lists of selected files may be saved and recalled later. Last visited folders are memorized and can easily be revisited. See Figure 1, for a snapshot of ***TableButler's ***file selector.

### File merging

**Figure 2:**
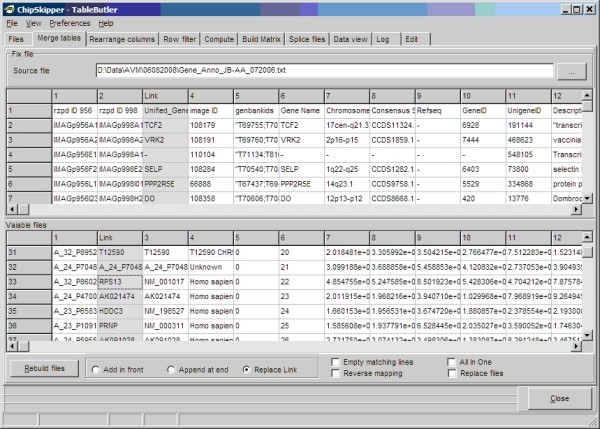
**Table merging: Top panel shows a preview of the table which will be inserted into all selected data table files**. Bottom panel shows a preview of the first from the selected files. Grayed columns indicate the selected columns containing the common keys used to identify identical lines. "Replace link" is selected: The whole data line in the Fix file is inserted in all selected files replacing the link-key.

Data tables are merged by combining rows from different tables, which contain the same identifier in a specified key column. This is a typical task when renewing or extending the annotations for all genes from a microarray with a gene index list. What sounds trivial for a few genes becomes challenging when re-annotating 140,000 features from a filter array with 50,000 gene annotations from Genbank or ENSEMBLE. One reference file may be inserted into all selected files. Vice versa, all matching genes from the list of selected files are assembled against the reference. See Figure 2, for a snapshot of ***TableButler's ***table merger.

### Column rearrangement

**Figure 3:**
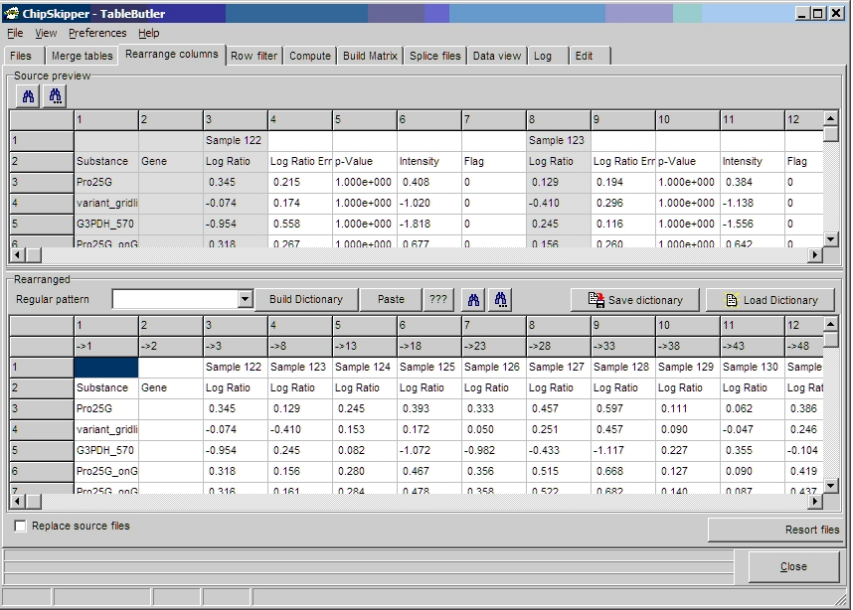
**Column rearrangement: The top panels show a preview of the source table**. Grayed columns indicate already selected columns. The bottom panel shows a preview of the result table. In the header column numbers from the source file are shown.

During statistical analysis or clustering it might be handy to change column order, e.g. to group experiments. Also, when submitting microarray data to databases not all data columns from the raw hybridization data tables are required. Published combined data matrices may contain several thousand data columns [11]. With ***TableButler***, one can easily reorder or reduce the columns in all selected data files in a batch. The re-order pattern can be comfortably set-up in a drag-and drop manner. First, one clicks the column in a source preview, and then one clicks the destination column in a result-preview for each required column. For large data files, regular expressions can be used to generate the pattern for hundred or thousands of columns. In addition, lists of externally generated column names may be used for reordering. See Figure 3, for a snapshot of ***TableButler's ***column rearranger.

### Row filtering

Not all rows (features) from a hybridization file are required or suited for subsequent statistical or cluster analysis. Spotting controls or spike-in genes for quality tracking of the wet-lab processing steps (RNA extraction, amplification, labeling, etc.) do not contribute any biological information for the study. Low quality genes can increase the signal noise in the statistical tests. Row filtering can be used to remove thus data rows from the data. Rows may be filtered upon text or numerical content of a single data column. Several filters (e.g. remove all genes containing "control" in the gene's description and quality flag <>"Pass") may be combined in a single run.

**Figure 4:**
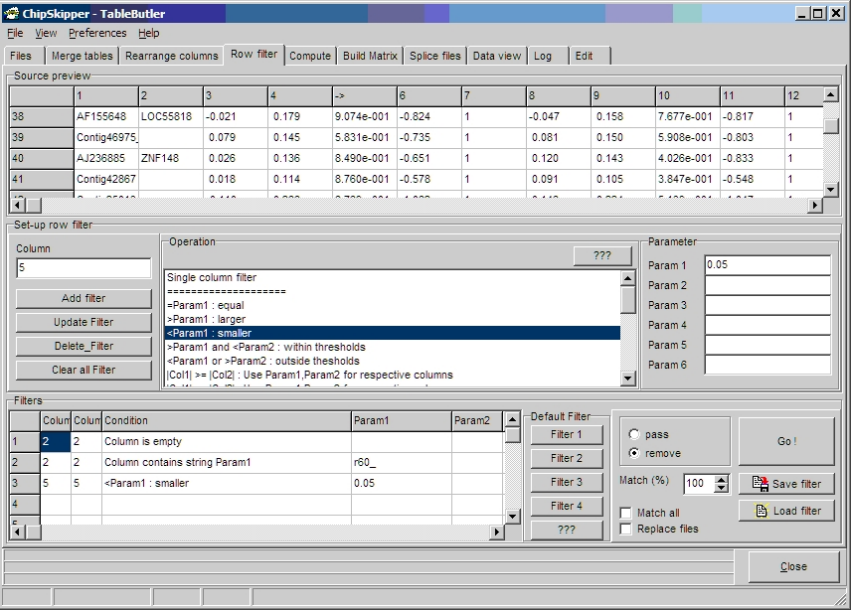
**Row filter: Top Panel shows a preview of the source data file**. Middle panel is used to set up the filter by choosing the type of filter and filter parameters. The buttons add a single set-up filter to the filter list. Bottom panels show all defined single filters (here: remove all rows without gene symbol or with control) and all rows where p-values < 0.05. Up to 5 custom defined filter set may be assigned to one of the five Default filter buttons.

When filtering multiple files simultaneously, the single file's filter can be combined (AND, OR) and applied to all files, thus generating a consensus list of genes fulfilling all filter criteria from all data tables. See Figure 4, for a snapshot of ***TableButler's ***row filter.

### Compute

Often additional data values or data transformations may be useful or required before further analysis. TableButler offers a variety of simple arithmetic, textual and statistical functions that are applied to data values in each gene row:

• Simple arithmetic (e.g. add/subtract constants to data columns, Log2, Log10->log2 transform, change sign, invert numbers, column sums, differences and ratios)

• Basic statistics, (min, max, arithmetic/geometric mean, variance, standard deviation t-test, ANOVA),

• Spot coordinate transformation (Sub grid, Row, Column ->Metarow, Metacolumn, Row, Column and inverse),

• Basic normalization (mean/median centring/normalisation)

• Data imputation for missing values (constant, row average, hot deck, most similar)

• Replica averaging of replicated genes (using gene ids/names as replica indicator)

• Text functions (replace find, split text, split complicated text using regular expressions...)

• Date to number conversions

**Figure 5:**
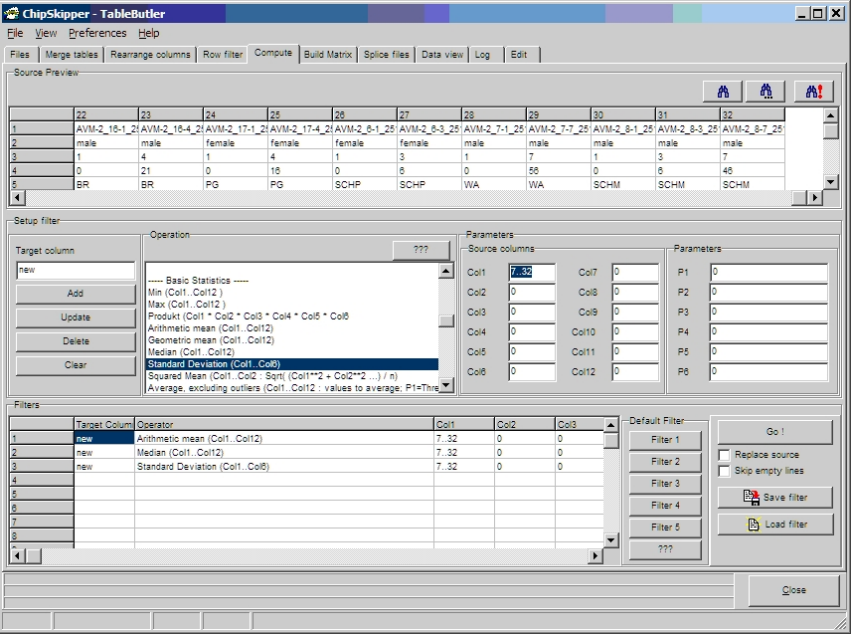
**Compute**. The top panel shows a preview of the source files, allowing to visually selecting the data columns for computations. The middle panel contains controls to set-up a single computation. From the operations list about 65 functions to perform various mathematical, statistical or text functions can be chosen. The bottom panel summarizes all defined operations. With the filter button, 6 customized predefined default computation sets may be loaded.

Similarly, a set of functions is available to re-compute data values based on mathematical operations applied to whole data columns. These functions serve mainly for simple normalization (mean/median centering/normalization, variance or quantile normalizations) across complete columns (= hybridizations). See Figure 5, for a snapshot of ***TableButler's ***column re-computer.

### Building a matrix

**Figure 6:**
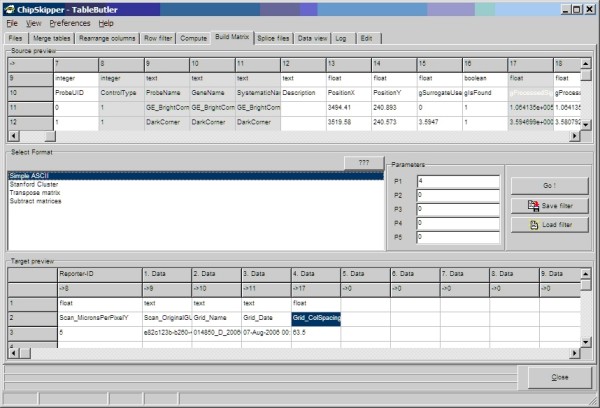
**Building an expression matrix**. Top panel shows a preview of the source data files. Grayed columns indicate those columns already selected for building a matrix. Middle panel allows defining file format and parameters. Bottom panel shows the structure of the result file.

Final adjusted and normalized ratios (from two color arrays) or intensities (from single color arrays) are combined into an expression matrix. ***TableButler ***allows to build generic expression matrices (i.e. on ratio/intensity per condition), or matrices with multiple data values per condition (e.g. collect ratio, single color intensities and quality values). In certain cases, transpositions of matrices may be required, which can also be performed. See Figure 6, for a snapshot of ***TableButler's ***matrix builder.

### Splice data tables

Here various functions to cut and combine data tables are found:

• Remove certain numbers of rows/columns from data files

• Append files (row or columns wise)

• Remove rows with replicated values in key columns (e.g. remove duplicated gene rows)

• Logically combine data files using a key column (Venn like analysis: get data rows from multiple files containing same genes in key columns using logical operators AND, OR, NOT, XOR).

### Data view

provides several graphs to visually inspect data with standard graphs:

Scatter plots, R/I-plots, quantile plots, Line graphs, Histograms, Box plots, Heat maps

In most cases, multiple operations (filtering, computations) may be combined. Some operations (e.g. t-tests) add multiple new columns to the data files. Here it is recommended to run such operations separately. Parameter sets for operations may be saved and recalled later, allowing standard processing of homologues data sets.

### Scripting

Furthermore, multiple filters may be combined in scripts, to realize complicated data workflows. An internal script editor allows composing scripts, supplying allowed script commands in nested pop-up menus. Scripts can be prototyped interactively, saving customized parameters for the single operations. Scripts may be loaded and executed manually or may be run automatically when ***TableButler ***is started with command line parameters.

### TableButler Server

***TableButler ***may even be run as server: A user-defined folder is watched. Any ***TableButler ***scripts dropped to this folder are automatically loaded and executed. The script folder or referenced data folder may be located on shared network resources.

## Results and discussion

***TableButler ***is a native Win32 application implemented with Borland's Delphi 5 and runs on Win32 operation systems (e.g. Win98, NT, 2000, XP, Vista). It does not require any additional supporting programs or libraries. ***TableButler ***can be copied to any computer with basic user privileges.

TableButler was applied in several collaborative research projects for preprocessing of gene expression data from large format filter arrays (140000 and 76000 features on filter macro-arrays [12]), custom spotted c-DNA microarrays (56000 features, [13-19],) and commercial Affymetrix (44000 features [20]).

For more details about ***TableButler's ***functionality and usage, visit the web page: .

## Conclusion

***TableButler ***is a monolithic Windows application. It is implemented to handle, join and preprocess batches of large tab delimited ASCII data files. The intuitive user interface enables scientists (e.g. biologists, clinicians or others) to setup workflows for their specific problems by simple drag-and drop like operations. Special knowledge about scripting languages (Perl, VBS, Java, SQL ...) is not required. TableButler can be executed without installation even from a memory stick. It does not require any supporting libraries or tools.

TableButler may be applied to any kind of tab delimited data table files: DNA expression data, Micro-RNA data, protein data, etc., even lists of telephone numbers or mp3-songs.

## Authors' contributions

CS designed, implemented ***TableButler ***and drafted the manuscript. AA und UW participated in design of functionality and user interface and applied ***TableButler ***in research projects. PH revised the manuscript. All authors read and approved the final manuscript.
